# Combined QTL Mapping across Multiple Environments and Co-Expression Network Analysis Identified Key Genes for Embryogenic Callus Induction from Immature Maize Embryos

**DOI:** 10.3390/ijms23158786

**Published:** 2022-08-07

**Authors:** Yun Long, Tianhu Liang, Langlang Ma, Peng Liu, Yun Yang, Xiaoling Zhang, Chaoying Zou, Minyan Zhang, Fei Ge, Guangsheng Yuan, Thomas Lübberstedt, Guangtang Pan, Yaou Shen

**Affiliations:** 1 State Key Laboratory of Crop Gene Exploration and Utilization in Southwest China, Maize Research Institute, Sichuan Agricultural University, Chengdu 611130, China; 2 Key Laboratory of Southwest China Wildlife Resources Conservation (Ministry of Education), College of Life Science, China West Normal University, Nanchong 637002, China; 3 Nanchong Academy of Agricultural Sciences, Nanchong 637000, China; 4 Department of Agronomy, Iowa State University, Ames, IA 50010, USA

**Keywords:** maize, immature embryo, embryogenic callus, QTL mapping, WGCNA, candidate gene

## Abstract

The ability of immature embryos to induce embryogenic callus (EC) is crucial for genetic transformation in maize, which is highly genotype-dependent. To dissect the genetic basis of maize EC induction, we conducted QTL mapping for four EC induction-related traits, the rate of embryogenic callus induction (REC), rate of shoot formation (RSF), length of shoot (LS), and diameter of callus (DC) under three environments by using an IBM Syn10 DH population derived from a cross of B73 and Mo17. These EC induction traits showed high broad-sense heritability (>80%), and significantly negative correlations were observed between REC and each of the other traits across multiple environments. A total of 41 QTLs for EC induction were identified, among which 13, 12, 10, and 6 QTLs were responsible for DC, RSF, LS, and REC, respectively. Among them, three major QTLs accounted for >10% of the phenotypic variation, including *qLS1-1* (11.54%), *qLS1-3* (10.68%), and *qREC4-1* (11.45%). Based on the expression data of the 215 candidate genes located in these QTL intervals, we performed a weighted gene co-expression network analysis (WGCNA). A combined use of KEGG pathway enrichment and eigengene-based connectivity (KME) values identified the EC induction-associated module and four hub genes (Zm00001d028477, Zm00001d047896, Zm00001d034388, and Zm00001d022542). Gene-based association analyses validated that the variations in Zm00001d028477 and Zm00001d034388, which were involved in tryptophan biosynthesis and metabolism, respectively, significantly affected EC induction ability among different inbred lines. Our study brings novel insights into the genetic and molecular mechanisms of EC induction and helps to promote marker-assisted selection of high-REC varieties in maize.

## 1. Introduction

Maize (*Zea mays* L.) is an important food and forage crop worldwide. Breeding superior varieties is the guarantee of high and stable yield in maize. Traditional genetic modification methods have a long cycle and are limited by natural variations [1], whereas transgenic breeding is able to break through these restrictions. In maize genetic transformation, the induction efficiency of embryogenic callus (EC) directly affects the transformation ratio of immature embryos [2]. Previous studies reported that EC induction from maize immature embryos was influenced by multiple factors, including genotypes, hormones, and compositions of induction media [3,4,5]. Notably, the capacity of EC formation showed serious genotype dependence when explants were incubated under the same condition [6]. Most elite lines in maize have low ratios of embryogenic callus induction (REC), which inhibits the development of maize transgenic breeding [7]. Identification of causal genes that control maize EC induction will contribute to the improvement of genetic transformation efficiency and transgenic breeding in maize. 

Genetic architecture for EC induction and regeneration abilities have been studied in several crops. For instance, QTLs controlling callus induction and another culture ability have been detected in wheat, while QTLs for callus induction rate and plant regeneration ability have been identified in rice [8,9,10]. In maize, a high-REC maize inbred line, 18–599 (R), and a low-REC line, R15, were used to construct an F2 population [11]. Using this population, five QTLs controlling callus induction efficiency were mapped on chromosomes 1, 3, 7, and 8, respectively, with the explained phenotypic variation ranging from 5.25% to 23.4% [11]. Eight QTLs responsible for tissue culture capacities were determined by using 239 recombinant inbred lines (RILs) derived from the maize inbred lines Huangzao4 (with a lower REC) and Mo17 (with a higher REC) [12]. These QTLs were located on chromosomes 2, 3, 5, 6, 8, and 9, which explained 4.78–14.02% of phenotypic variation [12]. A B73 near-isogenic line with a high REC was used for fine mapping of the QTLs responsible for EC response, and finally, a QTL was located in a 3053 kb region in bin 3.06 of chromosome 3 [13]. Based on the physical positions, these reported EC induction-related QTLs were located in different genomic regions of maize. Moreover, owing to the large intervals of these identified QTLs, no causal genes were identified based on the above QTLs. Conventionally, to further identify the functional genes involved in EC induction, QTL fine mapping should be conducted based on enlarged segregation populations and enhanced marker resolutions [14]. 

In addition to QTLs, several genes were reported to influence EC formation in maize, including *LEAFY COTYLEDON2* (*LEC2*), *BABY BOOM* (*BBM*), and *WUSCHEL2* (*WUS2*). The expression of *LEC2* was significantly upregulated during somatic embryogenesis in Arabidopsis explants induced in vitro, and a close link was observed between auxin and the *LEC2* activity [15]. The overexpression of maize *BBM* and *WUS2* genes produced higher transformation frequencies in many previously reported non-transformable maize inbred lines [16,17]. In a genome-wide association study of maize callus regeneration, the homologous gene of *WUS2*, *WOX2*, was confirmed as the candidate gene regulating the capacity of EC regeneration [18]. Our previous studies identified *ZmMYB138* and *ZmSAUR15*, which modulated EC formation by participating in the hormone signal transduction pathway [2,19]. *ZmSAUR15* encodes a small auxin-upregulated RNA, which negatively mediates the capability of maize EC formation by affecting IAA biosynthesis and cell division in immature embryo-derived callus [2]. *ZmMYB138* promotes the formation of EC through GA signal transduction in maize immature embryos [19].

Recently, a weighted gene co-expression network analysis (WGCNA) was developed to identify the hub genes in gene co-expression modules according to the gene expression patterns derived from transcriptome data [20]. WGCNA has been proven to facilitate the excavation of causal genes in larger QTL intervals of target traits, as it circumvents the requirement of further QTL fine mapping [14]. In this study, an IBM Syn10 DH population [21], which was constructed from the cross of maize inbred lines B73 and Mo17 and has a high recombination ratio, was used to identify the QTLs controlling EC induction from immature embryos of maize. All the gene models located in these QTL intervals were used to perform a WGCNA based on the RNA-seq data of four maize lines during the EC induction process. The EC induction-associated modules and hub genes were identified according to the KEGG pathway enrichment and eigengene-based connectivity (KME) values. Finally, gene-based association studies were performed to detect the intragenic variations influencing EC induction. Our study provided insights into understanding genetic and molecular mechanisms underlying EC formation in maize and developing molecular markers for improving the REC of immature maize embryos.

## 2. Results

### 2.1. Phenotypic Performances of EC Induction Traits under Three Environments

**Figure 1:**
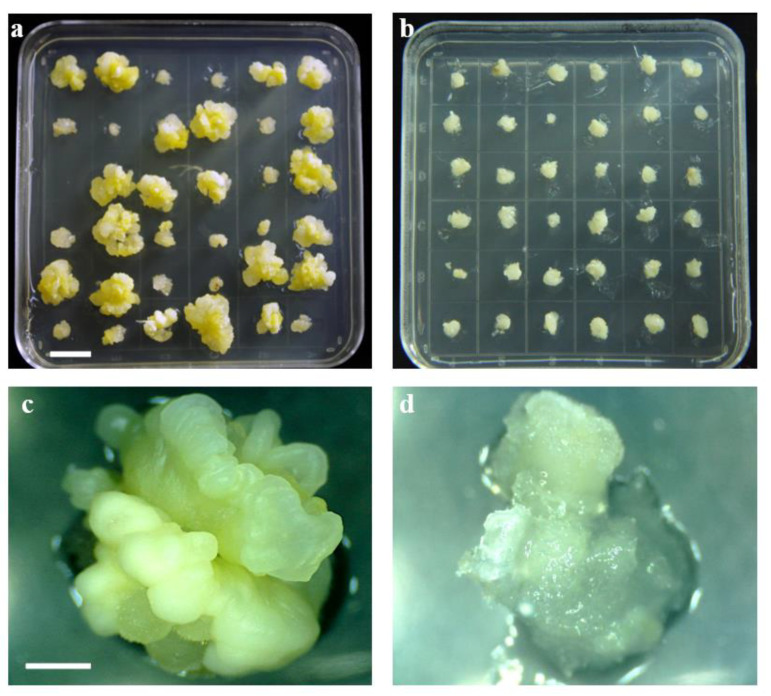
The calli induced from different doubled haploid lines in the IBM Syn10 DH population. (**a**,**c**) show the embryogenic callus (EC). (**b**,**d**) exhibit the non-embryogenic callus (NEC). Bar scale is 1 cm in (**a**,**b**) and 3 mm in (**c**,**d**).

To evaluate the phenotypic performances of EC induction capability among the IBM Syn10 DH population, we investigated the REC, DC, RSF, and LS for this population and its parents, B73 and Mo17, across three environments. The EC had a loose and fragile structure and formed globular nodules on the callus surface (Figure 1a,c), whereas non-embryogenic callus (NEC) presents a soft, transparent, and waterlogged structure (Figure 1b,d). After 30 d of incubation, the immature embryos of the parent B73 only formed NEC, with the REC = 0; approximately 5.79% (average across the three environments) of the Mo17 embryos formed EC (REC = 5.79%) (Table 1). However, the mean values of the rate of shoot formation (RSF), length of shoot (LS), and diameter of callus (DC) across the three environments were significantly greater in B73 than those in Mo17. 

**Figure 2:**
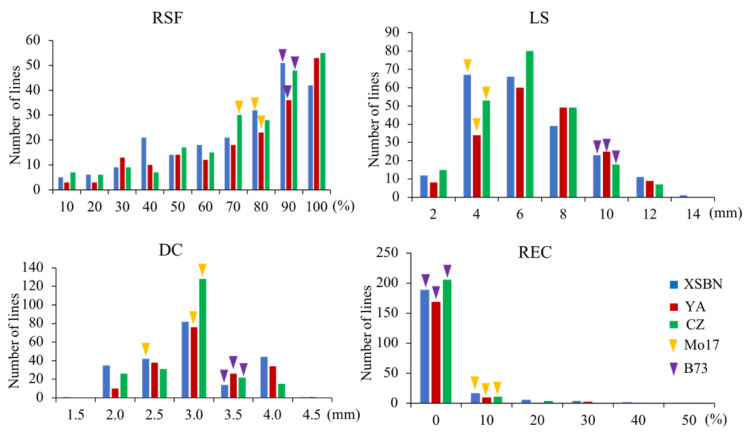
Phenotypic frequency distribution of the IBM Syn10 DH population across three environments. RSF, LS, DC, and REC represent the rate of shoot formation, length of shoot, diameter of callus, and rate of embryogenic callus induction. XSBN, YA, and CZ denote the environments Xishuangbanna, Ya’an, and Chongzhou, respectively. Mo17 and B73 show the two parents of the population.

Among the IBM Syn10 DH population, the REC ranged from 0 to 52.17%, 0 to 58.61%, and 0 to 56.48% under the three environments of Xishuangbanna (XSBN), Ya’an (YA), and Chongzhou (CZ), respectively, with the coefficients of variation (CVs) being 2.00, 2.90, and 2.67. However, most of these recombinant lines cannot form EC (with the REC = 0). The skewness and kurtosis of REC were > 1 under each environment (Table 1), suggesting that REC was an atypical quantitative trait (Figure 2). Interestingly, five recombinant lines displayed an REC > 10% (56.48%, 42.88%, 44.91%, 58.61%, and 12.04%) (Table 1 and Figure 1), exceeding their two parents, B73 and Mo17, in REC. It indicated that higher REC-associated alleles from B73 and Mo17 have been integrated into these recombinant lines. Meanwhile, the RSF, LS, and DC were continuously distributed among this population, with the respective CVs ranging from 0.35 to 0.37, 0.42 to 0.48, and 0.16 to 0.23 across different environments (Table 1). The absolute skewness and kurtosis values of these three traits were all smaller than 1 except for the RSF in the CZ environment, verifying that the RSF, LS, and DC generally conformed to normal distributions and were typical quantitative traits (Table 1 and Figure 2). 

Significantly negative correlations were observed between REC and RSF, REC and LS, and REC and DC in the three environments except for REC and RSF in CZ (Table 2). The correlation coefficients (CCs) of the three pairs of traits ranged from −0.105 to −0.342, −0.134 to −0.263, and −0.177 to −0.318 across the three environments, respectively. Furthermore, higher phenotypic correlations between DC and RSF, DC and LS, and LS and RSF were found across different environments, and the CCs ranged from 0.482 to 0.571, 0.612 to 0.715, and 0.692 to 0.737, respectively (*p* < 0.01). The broad-sense heritability (*H*^2^) of the four EC induction-related traits varied between 83.17 and 88.27% (Table 3), indicating that genetic factors played dominant roles in the formation of EC. Analysis of variance (ANOVA) revealed that the variances from genotype (G), environment (E), and genotype × environmental interactions (G × E) were all significant for the four traits (*p* < 0.01, Table 3). 

### 2.2. QTL Responsible for EC Induction

**Figure 3:**
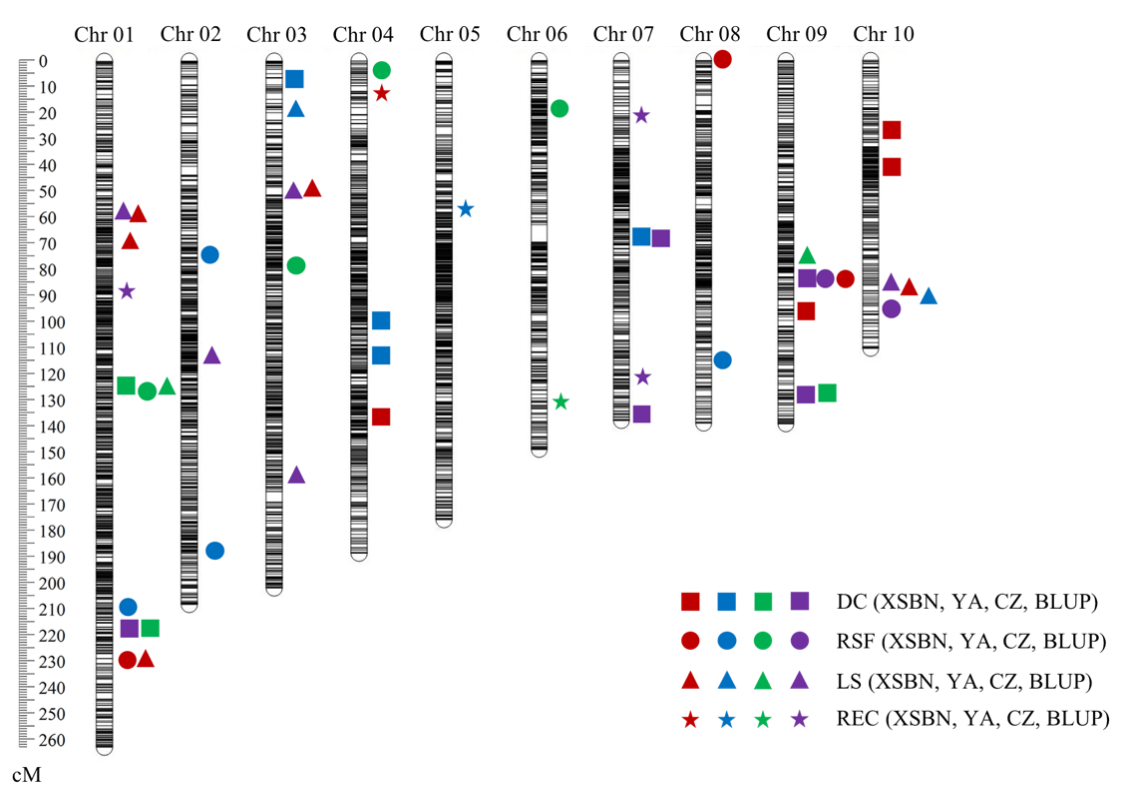
Chromosomal distributions of identified QTLs under three environments in this study. The red, blue, green, and purple represent Xishuangbanna (XSBN), Ya’an (YA), Chongzhou (CZ), and the best linear unbiased prediction (BLUP), respectively. Squares, circles, triangles, and pentagrams represent diameter of callus (DC), rate of shoot formation (RSF), length of shoot (LS), and rate of embryogenic callus induction (REC), respectively.

To detect the QTLs responsible for maize EC induction, we conducted linkage mapping by using the phenotype values collected from each environment and best linear unbiased prediction (BLUP) values. Finally, a total of 41 QTLs were identified for the four EC induction-related traits under the three environments and by using the BLUP values. These QTLs were distributed on 10 chromosomes, and the phenotypic variation explained (PVE) by a single QTL ranged from 4.01 (*qLS10-1* using BLUP) to 11.54% (*qLS1-1* in XSBN) (Table 4 and Figure 3). Three QTLs had the PVE > 10% and were thus considered as the major QTLs for EC induction ability. Furthermore, seven QTLs were individually simultaneously identified across different environments or across environment(s) and BLUP values, including three for DC, one for RSF, and three for LS. Among the 41 QTLs, 18 and 23 exhibited positively and negatively additive effects, respectively. The detailed information for these QTLs is presented below. 

#### 2.2.1. DC

Thirteen QTLs controlling DC were mapped to chromosomes 1, 3, 4, 7, 9, and 10, respectively. These individually explained 4.61–9.98% of the phenotypic variation and accounted for 28.15%, 30.32%, 22.32%, and 28.19% of the total DC variation under XSBN, YA, CZ, and using the BLUP values, respectively (Table 4). Among them, 11 QTLs presented positive additive effects, indicating that alleles from B73 contributed to the increase in DC (Table 4). Notably, *qDC1-2* and *qDC9-3* were separately co-detected across CZ and the BLUP model, whereas *qDC7-1* was repeatedly identified under YA and by using the BLUP values (Table 4 and Figure 3). 

#### 2.2.2. RSF

A total of 12 QTLs located on chromosomes 1, 2, 3, 4, 6, 8, 9, and 10 were identified for RSF (Table 4 and Figure 3). The PVE of a single QTL ranged from 5.33 to 9.97%, and the logarithm of odds (LOD) scores of these QTLs varied between 2.68 and 5.71 (Table 4). The total phenotypic variation explained by these RSF QTLs was 19.50%, 29.48%, 29.36%, and 14.03%, in XSBN, YA, CZ, and using BLUP, respectively (Table 4). One QTL (*qRSF9-1*) was co-identified across XSBN and BLUP, whereas the others were environment-specific QTLs (Table 4). In addition, eight QTLs on chromosomes 1, 2, 4, 8, and 10 showed negative effects, implying that alleles from Mo17 contributed to the increased RSF (Table 4). 

#### 2.2.3. LS

Ten QTLs affecting LS were detected on chromosomes 1, 2, 3, 9, and 10 across multiple environments and using BLUP. These QTLs explained 4.01–11.54% of the phenotypic variation, among which two major QTLs (*qLS1-1* and *qLS1-3*) had a PVE > 10%. Moreover, three QTLs were separately repeatedly detected under multiple environments or across environment(s) and BLUP (Table 4 and Figure 3). Two common QTLs (*qLS1-1* and *qLS3-2*) were individually repeatedly detected in XSBN and using BLUP, which explained 74–11.54% and 7.34–9.20% of phenotypic variation across different environments, respectively (Table 4). The other common QTL (*qLS10-1*) was simultaneously identified across XSBN, YA, and BLUP, with the PVE ranging from 4.01 to 6.98%. Moreover, nine QTLs showed negative additive effects, suggesting that the increased LS was mainly contributed by the alleles from Mo17 (Table 4).

#### 2.2.4. REC

Across the three environments and BLUP values, six QTLs for REC were mapped to chromosomes 1, 4, 5, 6, and 7, contributing 4.01–11.45% to the phenotypic variation (Table 4 and Figure 3). No QTL was repeatedly detected across multiple environments or across environment(s) and BLUP, suggesting that these identified QTLs were environment-sensitive QTLs (Table 4 and Figure 3). Notably, a major QTL (*qREC4-1*) was detected in XSBN, which explained 11.45% of the phenotypic variation (Table 4). In addition, among the six QTLs controlling REC, four QTLs on chromosomes 5, 6, and 7 displayed negative addition effects, indicating that the increased REC were mainly contributed by the alleles from Mo17 (Table 4).

### 2.3. QTL Clusters for Embryogenic Callus Induction-Related Traits

By comparing the physical positions among different QTLs identified in the present study, 12 were classified into four QTL clusters, which were located on chromosomes 1, 9 and 10, respectively (Table 5). Cluster A contained the QTLs controlling DC, LS, and RSF. Clusters B and D involved the QTLs for RSF and LS, whereas Cluster C included RSF and DC QTLs. Notably, each of the clusters contained the QTLs responsible for RSF, probably due to the high phenotypic correlation between RSF and each of DC and LS (Table 2 and Table 5). These universally observed clusters among DC, LS, and RSF reflected the pleiotropic effects of these QTLs on DC, LS, and RSF during the process of EC induction in immature embryos. However, the QTLs affecting REC were not included in any of clusters, which was consistent with the observation of the negative correlation between REC and each of DC, LS, and RSF. Collectively, these results suggested that the casual genes responsible for REC were independent from those for DC, LS, and RSF (Table 2 and Table 5). 

### 2.4. Validation of QTL Intervals

Reliability of one of the detected QTLs (*qREC6-1*) was validated by evaluating co-segregation between genetic variations in the QTL interval and REC phenotype using six high-REC lines (Mo17 and five other lines with REC >10%) and six low-REC lines (REC approximately 0). A total of 10 fragments covering 1035–3300 nucleotides in *qREC6-1* were amplified by PCR among these 12 lines. In fragment 1 (F1), only one SNP (G/A) was found; all six high-REC lines contained the G-allele, and the six low-REC ones contained the A-allele (Figure S1). In F2, three SNPs (G/A, T/C, T/C) were identified, and they formed two haplotypes (GTT and ACC). The six high-REC lines had the haplotype GTT, whereas the six low-REC lines had the haplotype ACC (Figure S2). Moreover, we identified four SNPs in F3, one SNP and one InDel in F4, four SNPs in F6, and three SNPs in F7. These 12 lines displayed co-segregations between the REC phenotype and genotype of each fragment (Figures S3, S4, S6, and S7). For each of F5, F8, F9, and F10, the high-REC lines possessed the same allele/haplotype, whereas the low-REC lines had the other allele/haplotype, except for the line IBM182 (a high-REC line), which contained the same alleles as the six low-REC lines (Figures S5 and S8–S10). Combined, these results verified the high credibility of the QTLs detected in the present study. Moreover, these SNPs/InDels can be used to develop the molecular markers for improvement of REC in immature maize embryos.

### 2.5. Candidate Genes and Co-Expression Networks

**Figure 4:**
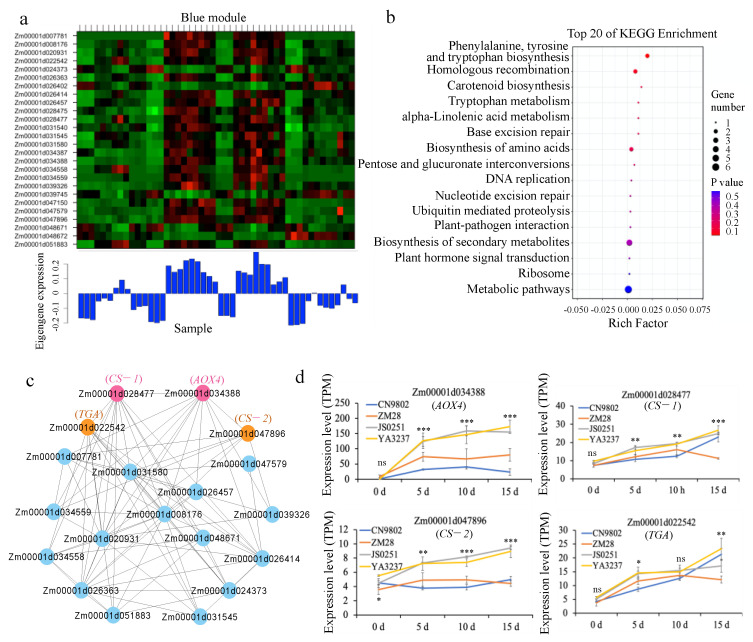
Gene co-expression network and hub genes responsible for maize embryogenic callus induction. (**a**) Eigengene expression pattern of the embryogenic callus induction-associated module (blue). Upper panel shows the gene expression heat map; lower panel shows the eigengene expression histogram. (**b**) Top 20 enriched pathways in blue module. (**c**) Co-expression network of hub genes. The four hub genes are shown in pink and orange. (**d**) The expression patterns of the four hub genes during embryogenic callus induction. CN9802, ZM28, JS0251, and YA3237 represent four different maize inbred lines. The significance of difference between each pair of these lines was estimated by using a *t*-test (2-tailed), with n = 3 (three biological replicates). *, **, and *** represent significant difference at *p* = 0.05, *p* = 0.01, and *p* = 0.001 levels, respectively, at least between one pair of the four lines, for each stage. ns = not significant. The bars show the standard deviation (SD).

To construct the co-expression networks involved in EC induction, we carried out a WGCNA for candidate genes responsible for EC induction capability. A total of 215 gene models were located in the confidential intervals of detected QTLs, based on the B73 v4 genome. Among them, 74, 58, 87, and 14 genes were responsible for DC, RSF, LS, and REC, respectively (Table S1). Our previous study performed transcriptome sequencing for four maize lines (CN9802, ZM28, JS0251, and YA3237) during the EC induction process. According to the transcriptome data, 122 of these 215 genes were differentially expressed at 5 d, 10 d, and/or 15 d after EC induction culture in at least one line, with the |log_2_(fold change)| > 1, and Q < 0.05. We then conducted a WGCNA using the expression data of the 122 genes. Finally, these 122 genes were grouped into six co-expression modules, namely green (14 genes), turquoise (32 genes), brown (24 genes), yellow (15 genes), blue (26 genes), and red (11 genes) (Table S2). Remarkably, KEGG enrichment analysis showed that the genes in the blue module were enriched in the phenylalanine, tyrosine and tryptophan biosynthesis pathway (Zm00001d028477 and Zm00001d047896), tryptophan metabolism pathway (Zm00001d034388), and plant hormone signal transduction pathway (Zm00001d022542) (Figure 4a,b). Since plant callus induction was extensively reported to correlate with tryptophan biosynthesis and metabolism, as well as hormone signal transduction [22,23,24], the blue module was thus considered as the EC formation-associated module in the present study. The above four genes were accordingly taken as the hub genes affecting EC induction, which showed high KME values (0.87–0.92) in the blue module (Figure 4c and Table S2). Generally, the four hub genes displayed increasing expression at each EC induction stage when compared with 0 h, verifying their response to EC induction (Figure 4d).

### 2.6. Hub Gene-Based Association Mapping

To further investigate whether these variations within the hub genes affected the REC among different maize lines, we performed hub gene-based association mapping by combining the phenotypes and genotypes of 75 lines with distinct genetic backgrounds. The maize lines consisted of the Tropical, Stiff Stalk (SS), and non-Stiff Stalk (NSS) germplasms previously genotyped using a maize 56K SNP Array (Zhang et al., 2016). Cluster analysis based on the genotypes showed that these 75 lines were classified into six different subgroups with 1–19 lines in each subgroup (Figure S11). The DC, RSF, LS, and REC of the lines were collected under three environments in our previous study [2]. The genotypes of these lines were obtained using PCR amplification and SNP/InDel calling for the four genes in the present study. After removing variants with missing rate > 0.2 or heterozygous proportion > 0.3, we identified 42 variants (42 SNPs) in Zm00001d028477, 10 variants (10 SNPs) in Zm00001d047896, 50 variants (48 SNPs and 2 InDels) in Zm00001d034388, and 44 variants (41 SNPs and 3 InDels) in Zm00001d022542 (Table S3). Association mapping revealed that six SNPs were significantly (*p* < 0.05) associated with the REC, DC, RSF, and LS in Zm00001d028477, and 20 SNPs were significantly (*p* < 0.05) associated with the four traits in Zm00001d034388 (Table S4). Meanwhile, two separate SNPs, one in Zm00001d022542 and the other in Zm00001d047896, were significantly (*p* < 0.05) associated with LS (Table S4). Among the six significant SNPs in Zm00001d028477, three SNPs (LS: S1_36255280 and S1_36256420; RSF: S1_36256406) were intron-splicing or missense variants, whereas the other three SNPs (REC: S1_36247233 and S1_36249669; DC: S1_36247066) were intron or synonymous variants (Table S4). For Zm00001d034388, nine SNPs (REC: S1_291663139; DC: S1_291664657; LS: S1_291666764 and S1_291663835; RSF: S1_291663009, S1_291665448, S1_291665505, S1_291664082, S1_291664657, and S1_291665851) were missense, 3′-UTR, or intron-splicing variants, whereas the other eleven SNPs were synonymous or intron variants (Table S4). Two SNPs located in Zm00001d022542 (LS: S7_179752120) and Zm00001d047896 (LS: S9_144619659) were also intron variants (Table S4). 

**Figure 5:**
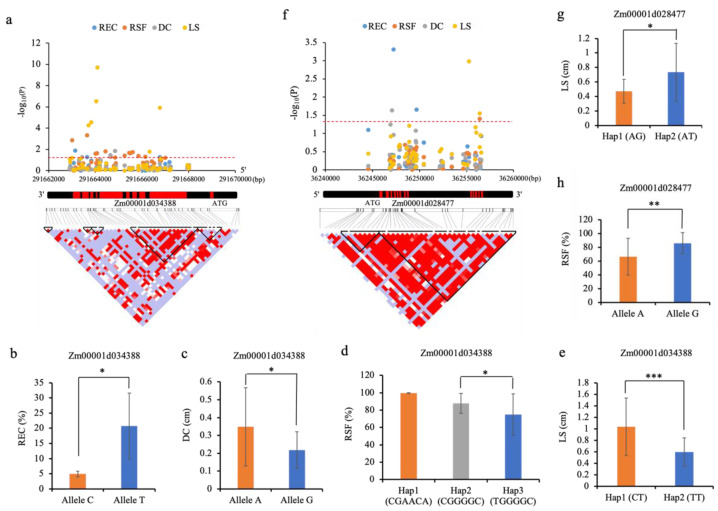
Hub gene-based association mapping. (**a**,**f**) Manhattan plots (top) and LD heat maps (bottom) of Zm00001d034388 and Zm00001d028477. The significance threshold is shown as a red dashed line (*p* < 0.05). The gene structure is shown in the middle. The exons are represented by filled red boxes. (**b**–**e**) Differences in (**b**) REC (rate of embryogenic callus), (**c**) DC (diameter of callus), (**d**) RSF (rate of shoot formation), and (**e**) LS (length of shoot) between alleles or haplotypes of Zm00001d034388. (**g**,**h**) Differences in (**g**) LS and (**h**) RSF between alleles or haplotypes of Zm00001d028477. Statistical significance is determined by two-tailed *t*-test: *, **, and *** denote significance at *p* = 0.05, *p* = 0.01, and *p* = 0.001 levels, respectively.

It is widely accepted that the missense and intron-splicing variants and those located in the UTR and promoter regions tend to generate alterations in amino acid or mRNA abundance [25,26]. We thus divided these 75 lines into different haplotypes by combining the types of significant variants (called effective variants in the present study) in each gene for a single associated trait. For Zm00001d034388, the effective REC-associated SNP classified these lines into two groups, and the group with the C-allele showed a significantly (*p* < 0.05) lower REC (5.50%) than that (22.60%) of the group with the T-allele (Figure 5a,b). Meanwhile, the effective DC-associated SNP divided these lines into two groups, and the group with the A-allele displayed a significantly (*p* < 0.05) higher DC (0.35 cm) relative to that (0.21 cm) of the group with the G-allele (Figure 5a,c). Furthermore, the six effective RSF-associated SNPs categorized these lines into three major haplotypes (Hap1: CGAACA; Hap2: CGGGGC; Hap3: TGGGGC), among which Hap2 had a significantly (*p* < 0.05) higher RSF (87.72%) than that (74.90%) in Hap3 (Figure 5a,d). According to the two effective LS-associated variants, these lines were divided into two major haplotypes (Hap1: CT; Hap2: TT), and Hap1 displayed a significantly (*p* < 0.001) larger LS value (1.04 cm) than that (0.59 cm) of Hap2 (Figure 5a,e). As for Zm00001d028477, the two effective LS-associated SNPs formed two major haplotypes (Hap1: AG; Hap2: AT), and the LS (0.73 cm) of Hap1 lines was significantly (*p* < 0.05) higher than that (0.47 cm) of Hap2 lines (Figure 5f,g). The effective RSF-associated SNP divided these 75 lines into two genotypes (A and G), and the RSF (51.23%) of the lines containing the A-allele was significantly (*p* < 0.01) lower than that (85.99%) of those containing the G-allele (Figure 5f,h). Collectively, these findings illustrated that the variants in Zm00001d028477 and Zm00001d034388 influenced the formation of EC among different maize lines.

## 3. Discussion

### 3.1. Use of IBM Syn10 DH Population for Mapping Embryogenic Callus Induction-Related Traits

Abundant phenotypic variations and high heritability estimates are both important to perform QTL mapping of complex traits [27]. In the present study, the parent B73 cannot form EC, whereas the REC of parent Mo17 ranged from 3.39–7.05% across three environments (Table 1). Notably, the REC of the IBM Syn10 DH population showed large variation, with the phenotypic value ranging from 0 to 52.17%, 0 to 58.61%, and 0 to 56.48% in XSBN, YA, and CZ, respectively (Table 1 and Figure 2). The broad-sense heritability estimates were larger than 80% for the investigated four traits, which contributed to the identification of the environmentally stable QTLs. A total of forty-one EC induction-related QTLs were detected, among which seven common QTLs were individually simultaneously detected across multiple environments or across the environment(s) and BLUP values, including three for DC (*qDC1-2*, *qDC7-1*, and *qDC9-3*), one for RSF (*qRSF9-1*), and three for LS (*qLS1-1*, *qLS3-2*, and *qLS10-1*). Moreover, high recombination rates and high-density markers in segregation populations facilitate fine mapping of the genetic loci controlling target traits [27]. The IBM Syn 10 DH population was obtained via six additional generations of open pollination on the IBM Syn4 RIL population and genotyped using Next-Generation Sequencing, resulting in a higher ratio of genetic recombination and a smaller genetic distance between two adjacent markers [18]. In the present study, the majority of the QTLs were mapped within < 0.2 Mb physical intervals, such as all the REC QTLs (*qREC1-1*, *qREC4-1*, *qREC5-1*, *qREC6-1*, *qREC7-1*, and *qREC7-2*). Compared to the larger intervals reported in previous studies on QTL mapping of EC induction [9,12,13], the smaller QTL intervals detected in our study facilitate further excavation of causal genes responsible for maize EC induction. 

### 3.2. Maize EC Induction Is Probably Controlled by a Few Major Genes

In contrast to LS, RSF, and DC, REC did not conform to a normal distribution among the IBM Syn 10 DH population (Figure 2), which was consistent with our observations in an association panel in our previous study [2]. It is widely accepted that quantitative traits show normal distributions only under the polygenic hypothesis [27]. However, the phenotype values will display a skewness among populations when the number of QTLs is small and there are a few QTLs with large genetic effects [28,29]. In the present study, only six QTLs were detected across different environments and using BLUP, including one major QTL with a PVE > 10% and five minor QTLs (Table 4 and Figure 3). Presumably, EC induction in immature maize embryos is controlled by a few major genes, which is helpful in the exploration of causal genes responsible for EC induction. 

### 3.3. QTLs for Embryogenic Callus Induction-Related Traits 

To date, most research on maize EC has focused on REC, callus weight, callus color, callus texture, callus proliferation ability, callus browning tendency, and callus regeneration ability [13,30,31,32]. However, adventitious shoots growing on immature embryos are considered to negatively influence the formation of EC in embryos [33]. Consistently, LS and RSF were significantly negatively associated with the REC across different environments in the present study (Table 2). Meanwhile, DC reflects the growth rate of EC [34]. Therefore, RSF, LS, and DC were taken as the novel traits for detecting genetic architecture of maize EC formation in the present study. 

The results of QTL mapping are susceptible to environmental influences [35], and QTL loci repeatedly identified in multiple environments are considered more convincing [36]. In this study, seven common QTLs (DC: three; RSF: one; LS: three) were separately simultaneously detected across multiple environments or across the environment(s) and BLUP (Table 4 and Figure 3). The QTLs controlling REC were separately mapped on chromosomes 1, 4, 5, 6, and 7, whereas none was repeatedly detected in multiple environments in this study (Table 4 and Figure 3). This implied a high environmental effect on REC in maize. A previous study reported two QTLs associated with the formation of embryo-like structures, which were located on the chromosomes 2 and 8, respectively [37]. The regions on chromosomes 1, 2, and 3 harbored the QTL controlling somatic embryogenesis in maize [38]. Five QTLs on chromosomes 1, 3, 7, and 8 were found to affect efficiency callus induction [11], whereas one QTL on chromosome 5 conferred EC initiation in maize [12]. Furthermore, a 23.9 Mb region on chromosome 3 was found to regulate embryogenic and regenerable tissue culture response in maize [13]. We compared the physical positions between the REC QTL identified in the present study and those reported previously; however, no overlap was found. One possible reason is that the mapping populations are different between our study and the previous studies, resulting in distinct variation effects on REC. 

### 3.4. Candidate Genes Involved in Embryogenic Callus Induction

Up to now, only a few genes have been proven to affect EC induction in maize, such as *ZmSAUR15*, *WUS2*, *BBM*, *LEC*, *SERK*, and *ZmMYB138* [15,16,17,19,39]. However, none of the above causal genes were located in the QTLs identified in the present study. The possible explanation was that no EC induction-associated variants existed within the genes among this IBM Syn10 DH population. In the present study, a combination of QTL mapping and WGCNA suggested that Zm00001d028477, Zm00001d047896, Zm00001d034388, and Zm00001d022542 were the hub genes in the EC induction-associated module. Among them, Zm00001d028477 and Zm00001d047896 encode chorismite synthases and participate in tryptophan biosynthesis. Zm00001d034388 was annotated as aldehyde oxidase 4, which is involved in tryptophan metabolism. Tryptophan acts as the precursor in auxin biosynthesis of plants [40], and the biosynthesis and metabolism of tryptophan exert impacts on auxin signal transduction [41]. A number of studies demonstrated the significance of auxin signal transduction on EC induction [42,43,44]. Our previous study revealed that the knockdown and overexpression of a key gene (*ZmSAUR15*) involved in auxin signal transduction improved and inhibited the induction of maize EC [2], respectively. Zm00001d022542 encodes a TGA transcription factor, a key factor in the pathway of salicylic signal transduction. Salicylic acid was extensively reported to affect callus culture and regeneration in different plant species [45,46,47]. The four hub genes showed increasing expression during the process of EC induction in the four lines, with the exception of Zm00001d047896 in the line CN9802. Gene-based associations revealed that significant variations within Zm00001d028477 and Zm00001d034388 affected EC induction-related traits among different inbred lines. These significant loci can be used to develop functional markers for improving the REC of immature maize embryos. 

## 4. Materials and Methods

### 4.1. Plant Materials and Field Trials

The IBM Syn10 DH population used for QTL mapping was derived from a cross between the lines B73 and Mo17, as described in our previous study [21]. In brief, this population was constructed through six additional generations of intercrossing on the IBM Syn4 population followed by haploid induction and chromosome doubling. Therefore, the IBM Syn10 DH population has a high genetic recombination frequency [22]. A total of 210 doubled haploid (DH) lines and their parents (B73 and Mo17) were investigated for the phenotypes of REC, RSF, LS, and DC. These lines were grown under three environments, namely XSBN (Yunnan province; 22.00° N,100.79° E; elevation, 553 m) in 2015, YA (Sichuan province; 29.59° N, 102.57° E; elevation, 516 m) in 2015, and CZ (Sichuan province, 30.30° N, 103.07° E; elevation, 560 m) in 2016. XSBN has a typical monsoon climate with a mean annual temperature of 21.3 °C in 2015, whereas YA and CZ have a monsoon-influenced humid subtropical climate with mean annual temperatures of 17.3 °C (YA, 2015) and 16.8 °C (CZ, 2016). A completely randomized block design was carried out with three replicates for each environment. Each line was grown in three rows with 14 plants per row. The row length was 3 m, and the space between two adjacent rows was 0.75 m. In each location, this population was managed using a standard cultivation practice for corn.

### 4.2. Immature Embryo Culture and Callus Induction

Three immature ears were harvested from the middle row per line at 12 d after self-pollination for each replicate. For each line, 108 immature embryos with 1.2–1.5 mm in length were collected from the middle sections of the three immature ears and evenly incubated among three Petri dishes with a modified N6 inducting medium [2]. These embryos were aseptically cultured at 28 °C in the dark for 30 days, with the scutella facing upward. A total of nine ears from three field repetitions were accordingly classified into three replicates in tissue culture.

### 4.3. Phenotype Investigation

The RSF, which was calculated as (number of embryos forming shoots/total number of inoculated embryos) × 100%, was investigated after 7 d of incubation. The LS (mm) and DC (mm) were measured after 10 d of incubation. The REC, which was equal to (number of embryos forming embryogenic calli/total number of inoculated embryos) × 100%, was scored after 30 d of incubation. The criteria for identification of EC were in accordance with the previous description [48]. 

### 4.4. Phenotypic Data Analysis

The average value of each trait among three replicates was taken as the phenotype value for each line. The SPSS software (version 26.0, IBM Corp., Armonk, NY, USA) was utilized to analyze the phenotypic data, including the range, mean value, CV, skewness, kurtosis, and correlation of each trait across three environments. Variance analysis incorporating genotype, environment, and genotype–environment interaction was conducted with the general linear model (GLM) in the SPSS 26.0 software. The broad-sense heritability (*H*^2^) of each trait was calculated following the previous description [49]. To reduce environmental effects on genotype, the BLUP values for the four traits were calculated using the lme4 package in the R software. The model is as follows: Phenotype~ (1|genotype) + (1|repeat % in % environment) + (1|genotypeand environment).

### 4.5. QTL Analysis

A bin map with high-density markers was constructed for the IBM Syn10 DH population in our previous study [50]. This bin map was approximately 11,198.5 cM in length, which contained 6618 bin markers with the average genetic distance between bin markers being 1.7 cM. The QTL IciMapping software version 3.0 [51] was applied to detect QTLs under three environments and using the BLUP values, based on inclusive composite interval mapping (ICIM). The testing window size of scan configuration was set to 10 cM, with a 2 cM walk speed. The significance threshold of LOD for QTL detection was set to 2.5, as described in the previous study [52]. Moreover, the QTLs explaining phenotypic variation > 10% were considered as major QTLs regulating EC induction. For each trait, QTLs with overlapped confidence intervals were assumed to be the same QTL [51]. The rule of QTL naming was as follows: q + trait + serial number of chromosome-serial number of the identified QTL. For example, in “qREC6-1”, “q” stands for QTL, “REC” is the abbreviation of induction rate of embryogenic callus, “6” represents chromosome 6, and “1” means the first QTL identified on chromosome 6. 

### 4.6. DNA Extraction and Variation Validation

The genomic DNA was extracted from the young leaves of B73, Mo17, 5 high-REC IBM Syn10 DH lines (IBM003, IBM097, IBM182, IBM270, and IBM304), and 5 low-REC IBM Syn10 DH lines (IBM009, IBM062, IBM144, IBM234, and IBM327) using the CTAB method [53]. In the qREC6-1 interval, 10 fragments with 1035–3300 nt in length were randomly selected and amplified using PCR. The amplified fragments were sequenced with Sanger sequencing. The sequencing results were aligned and analyzed with the SnapGene software (version 2.3.2, Insightful Science, USA, https://www.snapgene.com/). 

### 4.7. WGCNA 

Expression data of the gene models located in the detected QTL intervals were obtained from our RNA-Seq data of four maize lines (CN9802, ZM28, JS0251, and YA3237) during EC induction (Accession number: CRA007381, https://ngdc.cncb.ac.cn/gsa, accessed on 1 July 2022). Briefly, transcriptomes of the embryos were analyzed at 0 d, 5 d, 10 d, and 15 d after callus induction culture in the four maize lines. The clean reads were obtained by filtering the raw sequencing reads using the fastp software (version 0.23.1, Shifu Chen, Shenzhen, China) [54]. Gene expression values were calculated based on the clean reads mapped to the B73 RefGen_V4 genome and were subsequently normalized to transcripts per kilobase million (TPM) using an in-house script. Among the candidate genes obtained by QTL mapping, the differentially expressed genes (DEGs) were identified according to the absolute value of log_2_(fold change) between each induction stage (5, 10, and 15 d) and 0 d, and the threshold of DEGs was set to 1. The TPM of the DEGs were then subjected to a WGCNA using the WGCNA package in R software [20]. To detect the co-expression modules, WGCNA program parameters were set as follows: expression of variance data > 0; soft threshold = 11; max block size = 122; deep split = 2; min module size = 10; merge cut height = 0.2. In each of the co-expression modules, the Kyoto encyclopedia of genes and genomes (KEGG) pathway was analyzed on the GENE DENOVO platform (https://www.omicshare.com/tools/Home/Soft/pathwaygseasenior, accessed on 8 may 2021, GENE DENOVO Biotechnology Co., Ltd., Guangzhou, China). The co-expression network of the hub genes was drawn using the Cytoscape software (verision 3.9.1, Paul Shannon, Seattle, USA) [55].

### 4.8. Association Analysis

For each hub gene, the sequences of the gene body and its 2000 bp upstream were amplified by PCR in 80 randomly selected lines from our previously reported association panel [2]. PCR primers were designed in Primer 3 (version 0.4.0, Triinu Koressaar, Tartu, Estonia) based on the gene sequences in B73 RefGen_V4. Sequence alignment between the PCR-amplified sequences and the B73 genome sequence was conducted using the DNAMAN software (version 5.2.2, Lynnon Corp., Quebec, Canada) to detect sequence polymorphisms including single nucleotide polymorphisms (SNPs) and insertion/deletions (InDels). A GLM + PCA model in TASSEL software (version 4.0, Peter J. Bradbury, New York, USA) was used to detect associations between the phenotype values and polymorphism loci, with the significance threshold set as p = 0.05 [2]. Linkage disequilibrium (LD) decay between the markers was evaluated using HaploView software (Jeffrey Barrett, Cambridge, MA, USA). Haplotype identification was performed using the significant markers located in each hub gene.

## Figures and Tables

**Table 1:** Phenotypic performances of four traits in the IBM Syn10 DH population and its parents.

Trait ^a^	Environment ^b^	Parents (n = 6, *t*-Test)		IBM Syn10 DH Population					
		Mo17 ± SD	B73 ± SD	Range	Average	SD	CV%	Skewness	Kurtosis
RSF (%)	XSBN	81.34 ± 4.00	95.77 ± 0.64 **	0.00–99.07	67.64	24.99	0.37	−0.78	−0.42
	YA	81.82 ± 6.24	96.67 ± 1.96 **	0.00–100.00	70.84	25.53	0.36	−0.86	−0.25
	CZ	79.06 ± 2.35	94.44 ± 5.01 **	0.00–100.00	70.27	24.87	0.35	−1.02	0.26
LS (mm)	XSBN	4.0 ± 0.0	10.3 ± 0.6 **	0.0–15.0	5.5	2.65	0.48	0.70	0.20
	YA	4.3 ± 0.3	11.0 ± 1.0 **	0.0–12.0	6.0	2.54	0.42	0.26	−0.32
	CZ	5.0 ± 0.0	10.2 ± 1.8 **	0.0–11.7	5.3	2.29	0.43	0.38	0.13
DC (mm)	XSBN	2.7 ± 0.3	3.8 ± 0.3 **	1.5–4.3	2.9	0.66	0.23	0.34	−0.85
	YA	3.0 ± 0.0	3.8 ± 0.3 **	2.0–5.0	3.0	0.56	0.19	0.33	−0.50
	CZ	3.3 ± 0.3	3.8 ± 0.3	2.0–4.0	2.8	0.46	0.16	0.15	0.39
REC (%)	XSBN	6.94 ± 1.39	0.00 ± 0.00 **	0.00–52.17	4.39	8.79	2.00	3.08	10.54
	YA	7.05 ± 1.91	0.00 ± 0.00 **	0.00–58.61	2.74	7.95	2.90	4.48	22.56
	CZ	3.39 ± 1.23	0.00 ± 0.00 **	0.00–56.48	2.27	6.03	2.67	4.84	32.70

SD, standard deviation, CV, coefficient of variation. ** The difference is significant at the *p* = 0.01 level between B73 and Mo17. ^a^ RSF, LS, DC, and REC represent the rate of shoot formation, length of shoot, diameter of callus, and rate of embryogenic callus induction. ^b^ XSBN, YA, and CZ represent the three environments, namely Xishuangbanna, Ya’an, and Chongzhou, respectively.

**Table 2:** Phenotypic correlation between the traits across the three environments.

Environment	Trait	REC (n = 210)	SC (n = 210)	LS (n = 210)
XSBN	RSF	−0.310 **	0.531 **	0.692 **
	LS	−0.263 **	0.725 **	
	DC	−0.304 **		
YA	RSF	−0.342 **	0.482 **	0.693 **
	LS	−0.243 **	0.612 **	
	DC	−0.318 **		
CZ	RSF	−0.105	0.571 **	0.737 **
	LS	−0.134 *	0.639 **	
	DC	−0.177 *		

** and * represent significant correlation at the 0.01 and 0.05 levels by a *t*-test (2-tailed).

**Table 3:** Analysis of variance (ANOVA) and broad-sense heritability (*H*^2^) for four traits under three environments.

Trait	Source of Variation	df	Mean Square	Significance	*H*^2^ (%)
RSF	Genotype(G)	209	2092.792	<0.01 **	84.02
	Environment(E)	2	1136.991	<0.01 **	
	G × E	418	1147.495	<0.01 **	
	Error	1260	140.281		
LS	Genotype(G)	209	22.363	<0.01 **	85.64
	Environment(E)	2	40.006	<0.01 **	
	G × E	418	10.892	<0.01 **	
	Error	1260	1.067		
DC	Genotype(G)	209	1.076	<0.01 **	83.17
	Environment(E)	2	3.584	<0.01 **	
	G × E	418	0.633	<0.01 **	
	Error	1260	0.061		
REC	Genotype(G)	209	194.825	<0.01 **	88.27
	Environment(E)	2	369.202	<0.01 **	
	G × E	418	73.082	<0.01 **	
	Error	1260	13.689		

RSF, LS, DC, and REC represent the rate of shoot formation, length of shoot, diameter of callus, and rate of embryogenic callus induction. *H*^2^ represents the broad-sense heritability. ** denotes significance at *p* = 0.01 level by a *t*-test (2-tailed).

**Table 4:** QTLs controlling embryogenic callus induction-related traits across three environments.

Trait	Name	Env.	Chr.	Genetic Position (cM)	Physical Position (Mb)	LOD ^a^	PVE ^b^	ADD ^c^
DC	*qDC1-1*	CZ	1	125.84	191.425–191.600	3.02	7.10	−0.1174
	*qDC1-2*	CZ	1	217.21	286.200–286.300	2.61	5.73	0.1089
	*qDC1-2*	BLUP	1	217.49	286.400–286.550	3.55	4.64	0.0398
	*qDC3-1*	YA	3	9.85	2.700–2.800	3.19	6.59	−0.1322
	*qDC4-1*	YA	4	101.97	170.300–170.675	3.40	7.13	0.1407
	*qDC4-2*	YA	4	116.7	180.300–180.400	4.76	9.98	−0.1705
	*qDC4-3*	XSBN	4	138.78	210.875–211.000	2.85	5.30	0.1734
	*qDC7-1*	YA	7	71.29	132.275–132.525	3.21	6.62	0.1437
	*qDC7-1*	BLUP	7	72.01	132.800–132.900	4.84	6.40	0.0501
	*qDC7-2*	BLUP	7	140.95	174.175–174.300	3.42	4.61	−0.0391
	*qDC9-1*	BLUP	9	86.05	133.775–133.900	5.29	7.13	0.0504
	*qDC9-2*	XSBN	9	99.62	141.700–141.950	4.14	7.89	0.1858
	*qDC9-3*	CZ	9	131.38	152.200–152.300	3.92	9.49	0.1512
	*qDC9-3*	BLUP	9	132.23	152.600–152.700	4.06	5.40	0.047
	*qDC10-1*	XSBN	10	27.82	7.400–7.775	4.00	7.79	0.1936
	*qDC10-2*	XSBN	10	41.13	67.250–68.900	3.81	7.17	−0.184
RSF	*qRSF1-1*	CZ	1	127.16	192.525–193.650	4.29	7.37	−6.7656
	*qRSF1-2*	YA	1	211.45	280.975–281.100	3.46	7.59	−6.9403
	*qRSF1-3*	XSBN	1	230.48	290.700–290.800	3.21	6.49	−6.7815
	*qRSF2-1*	YA	2	75.79	32.650–33.175	2.68	5.33	−5.8722
	*qRSF2-2*	YA	2	190.64	232.500–232.600	4.75	9.95	−8.0007
	*qRSF3-1*	CZ	3	81.31	155.675–155.675	5.71	9.97	8.2628
	*qRSF4-1*	CZ	4	5.09	2.500–2.600	3.77	6.36	−6.4367
	*qRSF6-1*	CZ	6	17.13	13.600–13.725	3.31	5.65	6.0506
	*qRSF8-1*	XSBN	8	0.06	0.100–0.350	2.82	5.55	6.1132
	*qRSF8-2*	YA	8	115.14	169.300–169.400	3.30	6.61	−6.8164
	*qRSF9-1*	BLUP	9	86.7	133.900–134.000	4.49	6.89	2.655
	*qRSF9-1*	XSBN	9	87.33	134.000–134.100	3.73	7.46	7.3017
	*qRSF10-1*	BLUP	10	95.16	145.200–145.300	4.30	7.13	−2.8252
LS	*qLS1-1*	BLUP	1	58.26	23.675–23.925	6.83	9.74	0.3231
	*qLS1-1*	XSBN	1	60.77	24.975–25.225	7.70	11.54	0.946
	*qLS1-2*	XSBN	1	71.27	35.375–35.625	3.15	4.44	−0.5845
	*qLS1-3*	CZ	1	127.16	192.525–193.650	5.59	10.68	−0.8065
	*qLS1-4*	XSBN	1	231.2	290.800–290.900	3.51	5.03	−0.6832
	*qLS2-1*	BLUP	2	115.59	185.350–185.550	3.54	4.90	0.2473
	*qLS3-1*	YA	3	17.74	4.100–4.200	2.98	6.29	0.6625
	*qLS3-2*	BLUP	3	52.41	14.300–14.400	6.33	9.20	−0.8419
	*qLS3-2*	XSBN	3	52.68	14.650–14.850	5.14	7.34	−0.2805
	*qLS3-3*	BLUP	3	161.37	216.325–216.550	3.15	4.42	0.2312
	*qLS9-1*	CZ	9	77.71	117.825–118.200	3.27	6.05	−0.6074
	*qLS10-1*	BLUP	10	87.58	143.675–143.800	2.90	4.01	−0.2087
	*qLS10-1*	XSBN	10	91.16	144.600–144.700	3.96	5.90	−0.7004
	*qLS10-1*	YA	10	96.74	145.400–145.500	3.24	6.98	−0.741
REC	*qREC1-1*	BLUP	1	89.61	66.800–66.900	3.83	6.25	1.0291
	*qREC4-1*	XSBN	4	14.29	4.400–4.500	3.18	11.45	2.3818
	*qREC5-1*	YA	5	58.4	21.200–21.300	3.13	7.73	−2.2782
	*qREC6-1*	CZ	6	132.21	164.300–164.400	2.55	6.00	−1.5787
	*qREC7-1*	BLUP	7	23.37	5.600–5.700	2.51	4.01	−0.8092
	*qREC7-2*	BLUP	7	120.07	167.200–167.300	3.96	6.36	−0.9828

^a^ LOD represents the logarithm of odds score. ^b^ PVE denotes the phenotypic variance explained by each QTL. ^c^ ADD, additive effect values of the QTLs. Positive and negative values indicate that the increasing effect of phenotypic value is derived from the alleles of B73 and Mo17, respectively. Env. and Chr. represent environment and chromosome, respectively. XSBN, YA, and CZ denote the environments, Xishuangbanna, Ya’an, and Chongzhou, respectively. BLUP, the best linear unbiased prediction.

**Table 5:** QTL clusters for the embryogenic callus induction traits in the IBM Syn10 DH population across three environments.

QTL Cluster Number	Chromosome	Traits ^a^	QTL Names	Position (Mb)	Positive Alleles	Range of Explained Phenotypic Variation (%)
a	1	DC + LS + RSF	*qDC1-1*; *qRSF1-1*; *qLS1-3*	191.425–193.650	Mo17 + Mo17 + Mo17	7.10–10.68
b	1	RSF + LS	*qRSF1-3*; *qLS1-4*	290.700–290.900	Mo17 + Mo17	5.03–6.49
c	9	DC + RSF	*qDC9-1*; *qRSF9-1*	133.775–134.100	B73 + B73 + B73	6.89–7.46
d	10	RSF + LS	*qRSF10-1*; *qLS10-1*	143.675–145.500	Mo17 + Mo17 + Mo17 + Mo17	4.01–7.13

^a^ RSF, LS, and DC represent the rate of shoot formation, length of shoot, and diameter of callus, respectively.

## Data Availability

Transcriptome data used in this study are available in the Genome Sequence Archive (GSA) in National Genomics Data Center (NGDC) database with the accession number CRA007381.

## Supplementary Materials

The following supporting information can be downloaded at: https://www.mdpi.com/article/10.3390/ijms23158786/s1.
