# Supplementary material for: Combined QTL Mapping across Multiple Environments and Co-Expression Network Analysis Identified Key Genes for Embryogenic Callus Induction from Immature Maize Embryos
Source: Int J Mol Sci. 2022 Aug 7;23(15):8786. doi: 10.3390/ijms23158786 (PMC9368897; doi:10.3390/ijms23158786)
Supplement: Supplementary file 1 [file ijms-23-08786-s001.zip › Figure S10.pdf]

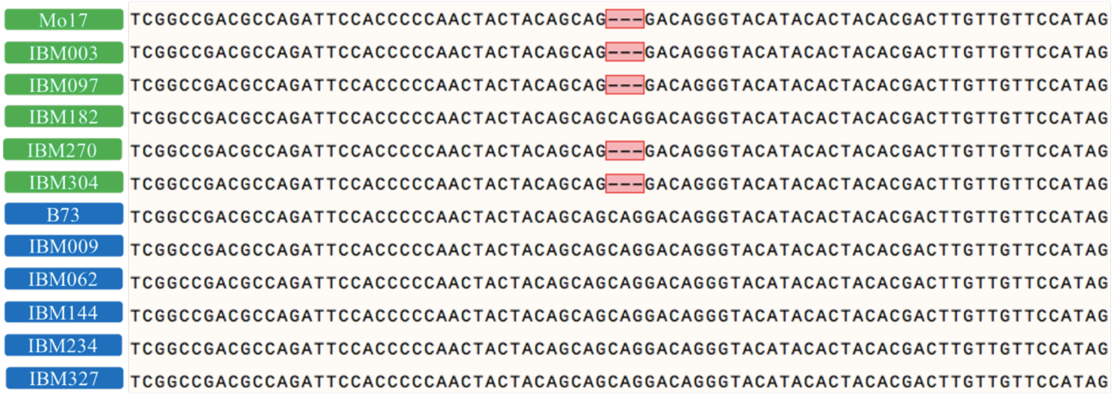

Figure S10 Sequence alignment of fragment 10 (F10) among parents and selected DH lines of the IBM Syn10 DH population. IBM003, IBM097, IBM182, IBM270, and IBM304 in green rectangular are high-REC lines, and IBM009, IBM062, IBM090, IBM144, IBM234, and IBM327 in blue rectangular are low-REC lines. The bases in red boxes represent the SNP locus in F10.
