# Supplementary material for: Combined QTL Mapping across Multiple Environments and Co-Expression Network Analysis Identified Key Genes for Embryogenic Callus Induction from Immature Maize Embryos
Source: Int J Mol Sci. 2022 Aug 7;23(15):8786. doi: 10.3390/ijms23158786 (PMC9368897; doi:10.3390/ijms23158786)
Supplement: Supplementary file 1 [file ijms-23-08786-s001.zip › Figure S11.pdf]

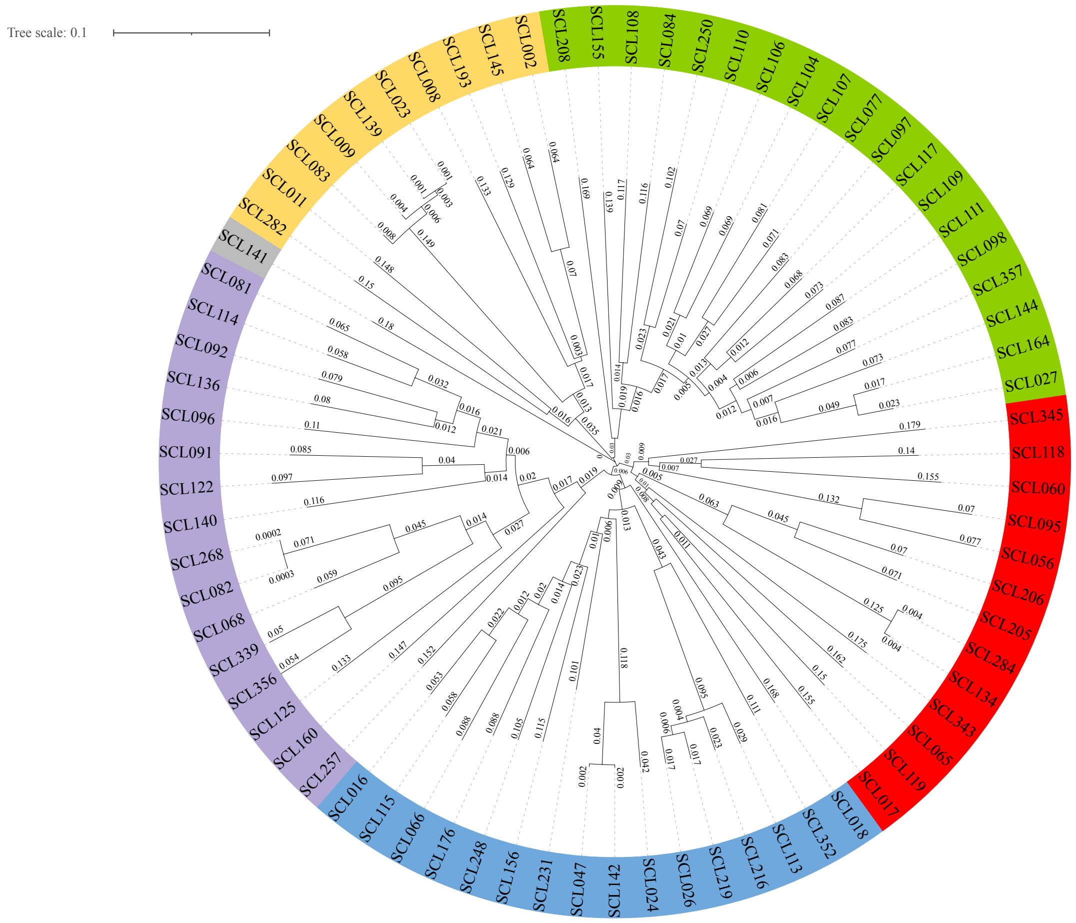

Figure S11 Phylogenetic tree of 75 maize inbred lines constructed using the Neighbor Joining method with 41,744 SNP markers. The name of each line was shown in the outer ring of the circle.
