# Supplementary material for: Combined QTL Mapping across Multiple Environments and Co-Expression Network Analysis Identified Key Genes for Embryogenic Callus Induction from Immature Maize Embryos
Source: Int J Mol Sci. 2022 Aug 7;23(15):8786. doi: 10.3390/ijms23158786 (PMC9368897; doi:10.3390/ijms23158786)
Supplement: Supplementary file 1 [file ijms-23-08786-s001.zip › Figure S5.pdf]

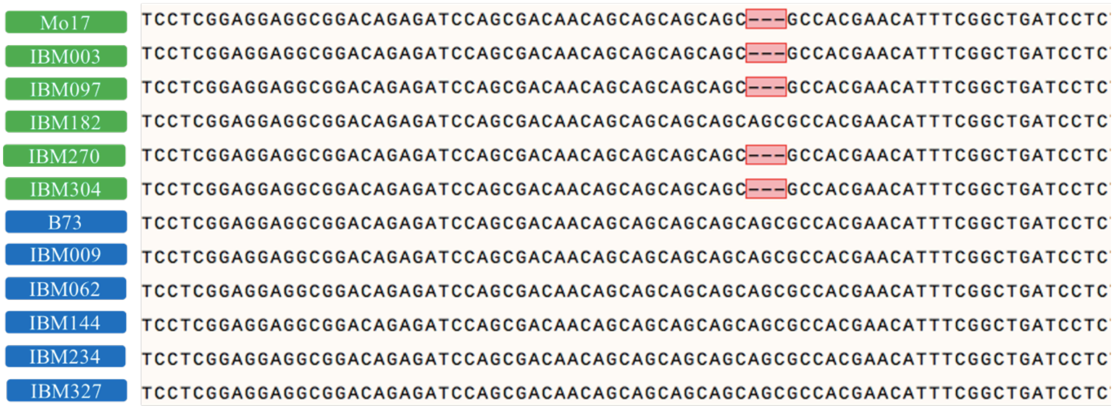

Figure S5 Sequence alignment of fragment 5 (F5) among parents and selected DH lines of the IBM Syn10 DH population. IBM003, IBM097, IBM182, IBM270, and IBM304 in green rectangular are high-REC lines, and IBM009, IBM062, IBM090, IBM144, IBM234, and IBM327 in blue rectangular are low-REC lines. The bases in red boxes represent the SNP locus in F5.
